# The burden of vestibular disorders among military health system (MHS) beneficiaries, fiscal years 2018–2019

**DOI:** 10.1371/journal.pone.0286798

**Published:** 2023-10-19

**Authors:** M. Aaron Sayegh, Amanda Banaag, Jessica Korona-Bailey, Cathaleen Madsen, Amanda Frank, Tracey Pérez Koehlmoos

**Affiliations:** 1 Center for Health Services Research, Department of Preventive Medicine and Biostatistics, Uniformed Services University of the Health Sciences, Bethesda, Maryland, United States of America; 2 The Henry M. Jackson Foundation for the Advancement of Military Medicine, Inc., Bethesda, Maryland, United States of America; 3 Department of Defense, Hearing Center of Excellence, Defense Health Agency, Joint Base San Antonio, San Antonio, Texas, United States of America; 4 zCore Business Solutions, Inc., Round Rock, Texas, United States of America; Universidade Federal de Sao Paulo/Escola Paulista de Medicina (Unifesp/epm), BRAZIL

## Abstract

**Introduction:**

Vestibular disorders affect an estimated 33 million adults and 3.5 million children and adolescents in the United States. Previous research relying on self-reported symptoms versus actual diagnosis has limited the ability to provide prevalence estimates for specific vestibular disorders at the population level. This study seeks to describe the burden of vestibular disorders among children and working-age adult beneficiaries in the Military Health System (MHS).

**Materials and methods:**

Using the MHS Data Repository (MDR), we conducted a cross-sectional study of all TRICARE Prime and Plus MHS beneficiaries aged 0 to 64 years from fiscal years (FY) 2018 to 2019. Study analyses included descriptive statistics of patient demographics and assessing the prevalence of vestibular disorders in pediatric and working-age adult beneficiaries.

**Results:**

Of the 5,541,932 TRICARE Prime/Prime Plus MHS beneficiaries, 52,878 (0.95%) had a diagnosis of vestibular disorder during fiscal years 2018 to 2019, of which 1,359 were pediatric and adolescents (aged 0 to 17 years) and 51,519 were working-age adults (18 to 64 years). Vertigo was the most common diagnosis in both age-group populations (11.46 per 1,000 working-age adults; 0.52 per 1,000 children and adolescents), with benign vertigo being the most prevalent of the three diagnoses and occurring at a seven times higher rate in adults versus pediatric and adolescents.

**Conclusions:**

This study demonstrates the effectiveness of using medical claims data to estimate prevalence compared to self-reported survey data and supports prevalence estimates of vestibular disease in <1% of children overall, but indicate much higher prevalence for adolescents.

## Introduction

Vestibular disorders affect an estimated 33 million adults in the United States (U.S.) [1] with the National Health and Nutrition Examination Survey (NHANES) reporting that 35% of U.S. adults ages 40 and older had evidence of balance dysfunction [2–4]. In children and adolescents an estimated 3.5 million [5] are affected with prevalence variance ranging between 0.7 to 15% [2–4]. These estimates mainly are derived from national health surveys that rely on self-report dizziness symptom screening and basic postural stability tests to quantify the burden of vestibular disorders among the population [2–4]. This rudimentary methodology is limited in providing the granularity necessary to assess the prevalence of specific vestibular disorders. These disorders are difficult to describe epidemiologically due to the low prevalence within the population and the lack of specificity to capture cases using the established survey methods [2–4].

The advent of the electronic medical record presents an alternative method for assessing the relative frequency of vestibular disorders. Specifically, within the Department of Defense (DOD), the Military Health System (MHS) offers a large database that is rich with the diagnostic characteristics for a socio-demographically diverse population of 9.6 million universally insured military and civilian beneficiaries. An estimated 18% of those individuals are on active duty; the remaining majority of beneficiaries are dependents and retirees [6–8]. MHS beneficiaries may receive services through the direct care (DC) system, via providers at military treatment facilities (MTFs), or the private-sector care (PC), via providers at civilian facilities through the TRICARE benefit [6–8]. TRICARE does not include care delivered in theater or through the Veteran’s Health Administration [6–8].

Altogether, analyzing the population trends within the MHS population may prove useful in assessing health status and diagnosis prevalence for the greater U.S. population. The objective of our study is to describe the burden of vestibular disorders among children, adolescents, and working age adult beneficiaries in the MHS. Examining the distribution of specific vestibular disorders could help to improve awareness of vestibular condition, access to health care, and drive future research initiatives. To the best of our knowledge, this study is one of the first to use healthcare claims data to assess the prevalence of vestibular disorders within the U.S. population.

## Methods

Using TRICARE claims data in the MHS Data Repository (MDR) we conducted a cross-sectional study of all TRICARE Prime and Plus MHS beneficiaries aged 0 to 64 years from fiscal years (FY) 2018 to 2019 (October 1, 2017 to September 30, 2019). We excluded beneficiaries aged 65 and older as Medicare becomes the primary insurance payer for people in this age group. The MDR contains all encounter and claims data for MHS beneficiaries, including records for approximately 1.6 million pediatric and adolescents aged 0 to 17 years and approximately 8 million adults aged 18 to 64 years [6–8]. Examination of the MDR healthcare claims data allows for a comprehensive understanding of which diseases and injuries are affecting the MHS and the severity of those impacts by gender, age, race, and service region.

Using the International Classification of Diseases, tenth revision (ICD-10), Clinical Management diagnostic codes, we identified all beneficiaries in the MHS study population with vestibular disorder diagnoses (H81.0-H81.9, H83.0-H83.3) during fiscal years 2018 and 2019. Patient demographic data such as gender, age, race, the patient or sponsor’s rank, and branch of service, were extracted from their beneficiary enrollment records, or at the time of diagnosis for those patients with a vestibular disorder. Rank was used as a proxy for socioeconomic status, as described in previous studies [9, 10]. In the MHS, data on race are missing for approximately 90% of dependent beneficiaries (i.e. children and spouses), but is a mandatory requirement for active duty personnel. To address missing race in our pediatric and adolescent population, we conducted relational imputation by using the known race of the sponsor for the unknown or missing child’s race [11].

### Analyses

We estimated the prevalence of vestibular conditions among the pediatric and adolescent beneficiaries (ages 0 to 17 years), and in working-age, adult beneficiaries (ages 18 to 64 years). Descriptive statistics such as mean age and the frequency distribution of sex, race, and military rank were estimated. All analyses were conducted using SAS 9.4 (SAS Institute, Inc, Cary, NC). The data were de-identified with no personal information provided in the data set. Informed consent was not required. This study was found exempt from the inclusion of human subjects by the Institutional Review Board of the Uniformed Services University of the Health Sciences.

## Results

Of the 5,541,932 TRICARE Prime/Prime Plus MHS beneficiaries, 52,878 (0.95%) had a diagnosis of vestibular disorder during fiscal years 2018 to 2019. Of those with vestibular disorders, 1,359 (2.6%) were pediatric and adolescents, aged 0 to 17 years, and 51,519 (97.4%) were working-age adults, aged 18 to 64 years. Among both pediatric and adolescent and adult beneficiaries, the mean age of patients with a vestibular disorder diagnosis was older than the mean of the total population (11.9 years compared to 7.4 years for the pediatric and adolescent beneficiaries; 45.3 years compared to 35.7 years for the working age adult beneficiaries) (Tables 1 & 2). When examined by age group, the frequency distribution of those with a vestibular diagnosis is skewed older relative to the total population with 39% between the ages of 15–17 years and 30% between the ages of 55–64 years, greater than their proportional representation in the total population (13% and 14%, for the pediatric and working adult age samples, respectively). The majority of patients diagnosed with a vestibular disorder were female (56.51% and 60.98%, for the pediatric and adolescent and working age adult population, respectively). The frequency distribution of working age adults who received a diagnosis of a vestibular disorder does not differ markedly by race from the total population. On the other hand, the proportion of pediatric and adolescent beneficiaries with a vestibular disorder who are not White is markedly less than their representation in the total population.

**Table 1:** Socio-demographic of the working age adult population, FY 2018–2019.

	Total Population (N = 3,935,399)	Diagnosed with Vestibular Disorder (N = 51,519)
**Mean Age (St. Dev)**	35.68 (13.70)	45.28 (12.68)
	**N (column %)**	
**Gender**		
Male	2186619 (55.56)	20102 (39.02)
Female	1748750 (44.44)	31417 (60.98)
**Age-group**		
18 to 24	1113052 (28.28)	3637 (7.06)
25 to 34	1032161 (26.23)	8421 (16.35)
35 to 44	661763 (16.82)	11006 (21.36)
45 to 54	583462 (14.83)	12813 (24.87)
55 to 64	544931 (13.85)	15642 (30.36)
**Race**		
White	1816044 (46.15)	22954 (44.55)
Black	501894 (12.75)	6530 (12.67)
Asian/Pacific Islander	166852 (4.24)	2442 (4.74)
American Indian/Alaska Native	28827 (0.73)	286 (0.56)
Other	104069 (2.64)	4310 (8.37)
Missing	1317683 (33.48)	14997 (29.11)
**Rank or Sponsor’s Rank**		
Junior Enlisted	1005182 (25.54)	3363 (6.53)
Senior Enlisted	2114800 (53.74)	26487 (51.41)
Junior Officer	453189 (11.52)	2900 (5.63)
Senior Officer	249114 (6.33)	4430 (8.60)
Warrant Officer	93039 (2.36)	1353 (2.63)
Other	20045 (0.51)	12986 (25.21)

**Table 2:** Demographics of the pediatric population, FY 2018–2019.

	Total Population (N = 1,606,569)	Diagnosed with Vestibular Disorder (N = 1,359)
**Mean Age (St. Dev)**	7.38 (5.42)	11.87 (4.76)
	**N (column %)**	
**Gender**		
Male	820563 (51.08)	591 (43.49)
Female	786000 (48.92)	768 (56.51)
**Age-group**		
0 to 4	587109 (36.54)	158 (11.63)
5 to 14	803904 (50.04)	666 (49.01)
15 to 17	215550 (13.42)	535 (39.37)
**Race**		
White	1099792 (68.46)	1003 (73.80)
Black	301603 (18.77)	188 (13.83)
Asian/Pacific Islander	106153 (6.61)	76 (5.59)
American Indian/Alaska Native	20250 (1.26)	18 (1.32)
Other	64210 (4.00)	58 (4.27)
Missing	14555 (0.91)	16 (1.18)
**Sponsor’s Rank**		
Junior Enlisted	209623 (13.05)	48 (3.53)
Senior Enlisted	1028308 (64.01)	621 (45.70)
Junior Officer	220515 (13.73)	123 (9.05)
Senior Officer	100761 (6.27)	95 (6.99)
Warrant Officer	46691 (2.91)	41 (3.02)
Other	665 (0.03)	431 (31.71)

Table 1 shows the proportion of the working age adults and pediatric and adolescent beneficiaries who have received a diagnosis of a vestibular disorder. Vestibular disorders are more prevalent in working-age adults compared to the pediatric and adolescent population. Benign vertigo was the most prevalent diagnosis in both working age adult and pediatric and adolescent populations, with estimates of 5.43 and 0.08 per 1,000 beneficiaries, respectively. The combined prevalence of labrynthitis, labrynthine dysfunction, Meniere’s Disease, noise effects on the inner ear, vestibular unspecified, vestibular neuronitis, and other vestibular dysfunction was 2.79 and 0.12 per 1,000 working-age adult and pediatric and adolescent beneficiaries, respectively.

Table 2 shows the frequency distribution of patients by number of concurrent vestibular diagnoses during the study period for both working age adult and pediatric and adolescent MHS beneficiaries. For the patients with a vestibular diagnosis coded during the two-year study period only 13.9% of the adults and 8.9% of the pediatric and adolescents had multiple vestibular conditions, with the majority only receiving one diagnosis.

## Discussion

This study is one of the first to estimate the prevalence of vestibular disorders in the pediatric, adolescent and adult patient populations using medical claims data in the U.S. We found that within the study population the prevalence of vestibular disorders was 1.56% in adults and 0.88% in children and adolescents. Our findings of vestibular disorder prevalence are consistent with estimates published using smaller clinical data sets of adult patients; however, when compared to national health survey estimates, the differences in prevalence are stark, with the survey results indicating that 35.4% of U.S. adults over the age of 40 [2, 3] and 23.3% of working age adults in the United Kingdom [2, 3] have vestibular dysfunction. Additionally, in children, our prevalence findings are at the lower end of previous estimates [2, 12, 13]. The differences between our prevalence findings and the results of health surveys may be rooted in the differences in criteria for categorization of patients and data collection methods. We specifically extracted H81.0-H81.9, H83.0-H83.3 ICD-10 codes from the MDR, which are specifically assigned to a patient by a medical provider if the patient suffering dizziness of vestibular origin. Our method of extraction did not capture patients who have dizziness or imbalance secondary to other factors such as orthostatic hypotension, lower extremity injury, or anxiety. Conversely, the national health survey method of categorizing patients does not take into consideration the origin of the dizziness or imbalance, instead the method only identifies the presence of a balance problem, be it self-reported or objectively found using a basic static balance measure. These methodological differences are likely the reasons behind our lower prevalence findings in both populations.

The appropriate use of vestibular ICD-10 diagnostic codes within the MHS may vary based on clinician specialization and experience. Unfortunately, many individuals with a true vestibular dysfunction are coded as: R42 dizziness and giddiness, H81.8 other disorders of vestibular function, or H81.9 unspecified disorders of vestibular function, which does not illustrate the medical diagnosis. Within the study population, benign paroxysmal positional vertigo (BPPV) was the most prevalent diagnosis for both adults and children. BPPV is a short-term episodic condition and is considered the most common type of vertigo experienced by adults [3, 14] and children [2, 12] within the U.S. Unsurprisingly in children, the next most frequent diagnoses, accounting to about 12%, were vestibular neuronitis and labyrinthitis, both commonly attributable to ear infections [2, 12]. Our analysis also found that 10% of children and 4.3% of adults were diagnosed with inner ear dysfunction due to noise effects. This high percentage raises concerns because noise induced ear damage is largely preventable and deserves further research.

The frequency distribution of vestibular diagnosis among MHS adult patients by age and gender reflects results of estimates from national surveys that use self-reports of dizziness and imbalance. We found that women comprised approximately 60.98% of adult patients with vestibular diagnoses, which is about 15% higher than the beneficiary population. These percentages are consistent with both a 2019 German study, which found 65.02% of those individuals with vertigo complaints were female [15] and epidemiological research that found women to be 2–3 times more likely to have a vestibular diagnosis [4]. The increased prevalence may be due to sex-based physiologic hormonal differences [16]; however, the reasons why women tend to report more vertigo remains unclear [17]. Interestingly, the gender differential in the adolescent population was lower, with females comprising 48.92% of the beneficiary population and only 56.51% of patients with vestibular disorder diagnoses. This result is in contrast to a Hungarian study that found males have higher prevalence of vestibular disorders prior to puberty; however, after reaching adolescence, females had a higher prevalence [18]. Further, our study found that 39.37% of vestibular diagnosis were among patients ages 15–17, which is large when compared to the percentage in the beneficiary population of 13.42%. The combining of children and adolescents in our study may underlay why the difference in prevalence due to hormonal changes at puberty was not found.

Consistent with previous literature we found that a greater proportion of the study population with vestibular diagnoses were older in age than the proportion of the beneficiary population. Individuals over the age 35 made up 76.59% of those patients with vestibular dysfunction compared to 45.45% in of the beneficiary population, with further analysis by decade, the greatest disparity was for patients aged 45–54, who comprised 24.87% of those patients with vestibular disorders, compared to only 14.83% of the total beneficiary population. Our results are consistent with these findings that as an individual ages, peripheral vestibular function decreases due to deterioration of vestibular hair cells [19]; however, we were not able to determine the specific reason for overrepresentation of vestibular disorders in those patients aged 45–54.

As in previous studies using the MDR, the current study uses Rank as a proxy for socioeconomic status [9, 10]. The proportions of Senior Enlisted (E5 and above), Warrant Officer, and Senior Officer (O4-O6) in the beneficiary population are approximately the same as in the study population. In contrast, Junior Enlisted (E1-E5) and Junior Officer (O1-O4) were under-represented among those patients with vestibular disorders. This underrepresentation may be due to the protective effects of younger age among junior vs. senior beneficiaries. Another possible explanation for the greater prevalence of vestibular diagnoses for those patients of higher rank is that early career service members may be less likely to report injuries due to military cultural stigmas against seeking medical care and those of higher rank may be more likely to report for retirement documentation purposes. The greatest difference in representation was among those patients of “Other” rank, who comprised 0.51% of the beneficiary population and over 25% of patients diagnosed with vestibular disorders. This difference may be due to the inclusion of older beneficiaries who no longer maintain a rank, but do not have sponsors. Similarly, in the pediatric and adolescent patients, the greatest difference was among “Other” rank in which 0.03% of the beneficiary population represented 31.71% of the diagnoses.

Race was not notable in the prevalence of vestibular disorders, though “Other” race, at 2.64% of the beneficiary population was overrepresented at 8.37% of patients with vestibular disorders; and “Missing,” at 33.48% of beneficiaries, was slightly underrepresented at 29.11% of patients with vestibular disorders. As with MHS adult beneficiaries, race was not notable in the prevalence of vestibular disorders in pediatric and adolescent patients. For example, 68.46% of the beneficiary population is White, but account for 73.80% of patients with vestibular disorders. Black race accounts for 18.77% of the beneficiary population, but only 13.83% of those patients with vestibular disorders. Whether the higher case counts among White pediatric and adolescent MHS patients are due to higher prevalence or higher rates of testing is unknown.

The results of this study demonstrate the effectiveness of using medical claims data to estimate prevalence compared to self-reported survey data. Survey data may be over-reporting estimates of vestibular disorders because they rely on self-reports of dizziness and imbalance, while medical claims could be underreporting given the difficulty in diagnosing. Further study is needed to determine whether results are true lower prevalence or show a lack of adequate diagnoses. Improved testing will likely decrease possible underreporting in medical claims data. Future research should continue to characterize the burden of vestibular disorders among this population and identify any racial disparities in diagnosis and care.

### Limitations

This study has several limitations. First, the use of claims data has the potential for coding errors and inadequate specificity for a condition. Second, this study is purely descriptive and did not assess causality of the conditions in question. Third, this study only assesses the frequency distribution of the vestibular disorders by one demographic variable at a time. The interaction effect of demographic characteristics on the distribution of the condition/s in question were not able to be assessed due to cell size limitations. Additionally, we were not able to fully describe beneficiaries in the “Other” rank category and “Other” race category which were disproportionately represented in the vestibular disorder population for both pediatric and adolescent and adult working age populations. Imputing race information may result in inaccurate race classification for study participants. While this method had been validated [20], it does not completely eliminate selection bias.

## Conclusions

Demographic characteristics of adult patients with vestibular disorders are consistent with previous studies from national surveys and prevalence estimates from clinical studies. These findings support prevalence estimates of vestibular disease in >1% of children overall, but indicate a much higher prevalence for adolescents. Further research should focus on equal opportunity for screening and diagnosis of children of all races. These results provide a detailed insight into the distribution of vestibular disorders among U.S. children and adults and inform efforts to surveille the broader category of communication disorders. Such knowledge has important implications for public awareness efforts of vestibular disorders in the MHS as well as in the U.S. population.
